# Brain regional susceptibility to tauopathy in individuals at risk for chronic traumatic encephalopathy

**DOI:** 10.1002/alz.71503

**Published:** 2026-06-16

**Authors:** Tim L. T. Wiegand, Anna Rubinski, Leonard B. Jung, Nicolai Franzmeier, Hector Arciniega, Parsa Ravanfar, Anna Dewenter, Michael L. Alosco, Yorghos Tripodis, Yi Su, Hillary Protas, Alexander P. Lin, Ofer Pasternak, Kewei Chen, Michael J. Coleman, Sylvain Bouix, Charles H. Adler, Laura J. Balcer, Charles Bernick, Jeffrey L. Cummings, Robert A. Stern, Eric M. Reiman, Martha E. Shenton, Michael Ewers, Inga K. Koerte, for the DIAGNOSE CTE Research Project

**Affiliations:** ^1^ Psychiatry Neuroimaging Laboratory, Psychiatry Department Mass General Brigham Academic Medical Centers Boston Massachusetts USA; ^2^ Harvard Medical School Boston Massachusetts USA; ^3^ cBRAIN, Department of Child and Adolescent Psychiatry, Psychosomatics, and Psychotherapy University Hospital Ludwig‐Maximilians‐Universität Munich Germany; ^4^ Computational Neurology, Department of Neurology Charité‐Universitätsmedizin Berlin Berlin Germany; ^5^ Computational Neurology Berlin Institute of Health Berlin Germany; ^6^ Institute for Stroke and Dementia Research (ISD) University Hospital, LMU Munich Munich Germany; ^7^ Department of Neurosurgery LMU University Hospital, LMU Munich Munich Germany; ^8^ Munich Cluster for Systems Neurology (SyNergy) Munich Germany; ^9^ Institute of Neuroscience and Physiology Department of Psychiatry and Neurochemistry, Mölndal and Gothenburg University of Gothenburg, The Sahlgrenska Academy Gothenburg Sweden; ^10^ Department of Rehabilitation Medicine NYU Grossman School of Medicine New York New York USA; ^11^ NYU Langone Concussion Center NYU Langone Health New York New York USA; ^12^ Melbourne Neuropsychiatry Centre, Department of Psychiatry The University of Melbourne and Melbourne Health Carlton South Victoria Australia; ^13^ Alzheimer's Disease Research Center and CTE Center Boston University Chobanian & Avedisian School of Medicine Boston Massachusetts USA; ^14^ Department of Neurology Boston University Chobanian & Avedisian School of Medicine Boston Massachusetts USA; ^15^ Department of Biostatistics Boston University School of Public Health Boston Massachusetts USA; ^16^ Banner Alzheimer's Institute Phoenix Arizona USA; ^17^ Center for Clinical Spectroscopy Department of Radiology Brigham and Women's Hospital, Harvard Medical School Boston Massachusetts USA; ^18^ Department of Radiology Brigham and Women's Hospital, Harvard Medical School Boston Massachusetts USA; ^19^ Department of Psychiatry Massachusetts General Hospital, Harvard Medical School Boston Massachusetts USA; ^20^ Department of Software Engineering and Information Technology École de technologie supérieure, Université du Québec Montréal Quebec Canada; ^21^ Department of Neurology Mayo Clinic College of Medicine, Mayo Clinic Arizona Scottsdale Arizona USA; ^22^ Department of Neurology NYU Grossman School of Medicine New York New York USA; ^23^ Department of Population Health NYU Grossman School of Medicine New York New York USA; ^24^ Department of Ophthalmology NYU Grossman School of Medicine New York New York USA; ^25^ Lou Ruvo Center for Brain Health Cleveland Clinic Las Vegas Nevada USA; ^26^ Department of Neurology University of Washington Seattle Washington USA; ^27^ Chambers‐Grundy Center for Transformative Neuroscience Department of Brain Health Kirk Kerkorian School of Medicine , University of Nevada Las Vegas Las Vegas Nevada USA; ^28^ Departments of Neurosurgery and Anatomy & Neurobiology Boston University Chobanian & Avedisian School of Medicine Boston Massachusetts USA; ^29^ German Center for Neurodegenerative Diseases (DZNE) Munich Germany; ^30^ Graduate School of Systemic Neurosciences Ludwig‐Maximilians‐Universität Munich Germany

**Keywords:** American football, CTE, dementia, functional connectivity, myelin levels, neurodegeneration, neuroimaging, repetitive head impacts, tauopathy, tau‐PET

## Abstract

**INTRODUCTION:**

Chronic traumatic encephalopathy (CTE) is a tauopathy linked to repetitive head impacts. Factors influencing brain regional susceptibility to tau deposition and spreading remain unclear.

**METHODS:**

We used three datasets: [18F]flortaucipir positron emission tomography (PET) in 157 former professional American football players and 53 controls (DIAGNOSE CTE); cortical myelin water fractions (MWF) in 50 healthy individuals (Myelin Water Atlas); and white matter (WM) tract MWF and functional connectivity (FC) in 100 healthy individuals (Human Connectome Project). We tested associations between tau‐PET uptake and covariance in football players and typical cortical gray matter (GM) MWF, WM tract MWF, and FC.

**RESULTS:**

Cortical regions with lower typical GM MWF showed higher tau‐PET uptake (*β* = −0.399, *p* = 0.001). WM tracts with lower typical MWF were associated with higher tau‐PET covariance (*β* = −0.238, *p* < 0.001). Higher typical FC was associated with higher tau‐PET covariance (*β* = 0.447, *p* < 0.001).

**DISCUSSION:**

In former football players at risk for CTE, regional susceptibility to tau deposition may be driven by low myelin and high FC.

## Highlights

- Key role of myelin levels and functional connectivity in brain regional susceptibility to tau.
- Methodological framework to model the pathomechanism of chronic traumatic encephalopathy (CTE) in vivo and non‐invasively.
- Insights into general characteristics of tau proteins.

## 1 BACKGROUND

Chronic traumatic encephalopathy (CTE) is a neurodegenerative disease associated with exposure to repetitive head impacts (RHI).^1^, ^2^, ^3^ It is a secondary tauopathy with three‐ and four‐repeat (3R/4R) tau isoforms^4^ and can only be diagnosed *post mortem* by neuropathological assessment.^5^ Diseased brains show an accumulation of hyperphosphorylated tau (p‐tau) in neurofibrillary tangles (NFTs).^1^, ^5^, ^6^ The progressive nature of CTE is characterized by a distinct spatial pattern of NFT progression across the brain.^3^, ^7^ In early stages, tau aggregates are localized predominantly in the frontal, temporal, and parietal cortex.^3^, ^7^ In later stages, tau often spreads irregularly across the cortex, diencephalon, basal ganglia, and brainstem.^3^, ^7^ The mechanisms behind the regional susceptibility to tau deposition and the pattern of tau spreading in CTE in vivo are not known.

Growing evidence from preclinical studies suggest prion‐like features of pathological tau that spreads via connected neurons.^8^, ^9^, ^10^, ^11^, ^12^ More specifically, tau proteins obtained from brain tissue of deceased CTE patients replicated in cultured cells after infecting them.^8^ Furthermore, in vitro and in vivo studies show trans‐synaptic tau spreading,^11^, ^13^ amplified by a higher activity shared between neurons.^14^, ^15^ One recent study demonstrated that following severe traumatic brain injury in humans, the affected brain tissue contains pathological tau protein that, when inoculated into the brains of mice, transmits and induces cognitive decline.^16^

In Alzheimer's disease (AD), another secondary 3R/4R tauopathy, several studies have investigated mechanisms of deposition and spreading of tau in the living brain using positron emission tomography (PET) tracers that bind to NFT, in combination with magnetic resonance imaging (MRI).^17^, ^18^, ^19^, ^20^ More specifically, cross‐sectional studies have investigated tau‐PET uptake covariance, defined as the correlation of tau levels between pairs of cortical regions. This measure has been interpreted as a proxy for tau spreading, as the spatial covariance patterns of tau‐PET uptake resemble the topology of functional brain networks. Such patterns are consistent with the hypothesis that tau spreads along structural and functional connections.^17^ We henceforth refer to this phenomenon as “correlated tau deposition”. Importantly, studies assessing spreading of tau longitudinally have shown similar associations with functional connectivity.^17^, ^18^, ^19^ Together, the studies showed that cortical regions with higher myelin water fraction (MWF), an MRI‐based measure of myelin, are less susceptible to tau deposition.^20^ In addition, higher white matter (WM) tract MWF showed more attenuated correlated tau deposition in connected cortical regions.^20^ On the other hand, higher functional connectivity between brain regions was associated with greater correlated tau deposition or longitudinally measured spreading of tau between these cortical regions.^17^, ^18^, ^19^ Associations between correlated tau deposition and functional connectivity were also observed in primary 4R tauopathies such as cortico‐basal degeneration and progressive supranuclear palsy,^21^ as well as in ageing.^19^ Biological substrates underlying the pattern of correlated tau deposition or tau spreading in CTE may be similar.

The aim of this study is to investigate what structural and functional factors lie behind the increased susceptibility of some brain regions to tau deposition and drive correlated tau deposition in a unique sample of former American football players exposed to RHI and at risk for CTE. To this end, we test associations between tau‐PET from football players with typical cortical and WM tract MWF as well as functional connectivity from healthy young adults. More specifically, tau‐PET in football player stems from the Diagnostics, Imaging, And Genetics Network for the Objective Study and Evaluation of Chronic Traumatic Encephalopathy (DIAGNOSE CTE) Research Project,^22^ and typical MWF and functional connectivity from the Human Connectome Project (HCP)^23^ and the Myelin Water Atlas.^24^ Similar to the studies in AD, we hypothesize (1) that cortical regions with lower gray matter (GM) myelin levels have greater tau deposition, (2) that cortical regions connected by WM tracts with lower myelin levels show more correlated tau deposition, and (3) that correlated tau deposition between cortical regions is associated with functional connectivity.

**RESEARCH IN CONTEXT**

1. **Systematic review**: It is not known what structural and functional factors lie behind the unique brain regional pattern of tau deposition and spreading in chronic traumatic encephalopathy (CTE). Research in Alzheimer's disease and four‐repeat tauopathies indicates that myelin levels and functional connectivity may be central.
2. **Interpretation**: In living former American football players at risk for CTE those cortical regions characterized by typically having lower myelin levels may be more susceptible to tau deposition. In addition, white matter tracts typically having lower myelin levels as well as stronger functional connections between brain regions may facilitate tau spreading.
3. **Future directions**: Future studies should validate the results by using longitudinal imaging data, improved tau‐positron emission tomography (PET) tracers, and repeating the analyses in individuals who receive a diagnosis of CTE *post mortem*.

## 2 METHODS

### 2.1 Samples

We used data from three research studies – one on former American football players, and two on healthy young adult samples. To study neurofibrillar tau deposition in former American football players exposed to RHI who are at increased risk for CTE, we assessed [18F]flortaucipir PET from the DIAGNOSE CTE Research Project.^22^ In addition, we used information on typical MWF from the Myelin Water Atlas^24^ and the HCP.^23^ These include neuroimaging data from healthy young adults, whose brains are likely fully developed and not impacted by age‐related or disease‐related factors.^25^ In the following, we provide information on each of the datasets used in this study.

#### 2.1.1 DIAGNOSE CTE

DIAGNOSE CTE is a multicenter, observational, cohort study that aims to identify in vivo biomarkers and diagnostic criteria for CTE.^22^ We recruited 180 former American football players (120 former professional players and 60 former collegiate football players), as well as 60 control participants without a history of neurotrauma and without cognitive or psychiatric symptoms.

The inclusion criteria for all study participants were: (1) male sex; (2) age between 45 and 74 years; (3) English as primary language; (4) no contraindications for neuroimaging or lumbar puncture; (5) consent to all procedures and willingness to have available a study partner (i.e., a spouse, a life partner, a close friend, or a family member). In addition, the former professional football players were required to have played a minimum of 12 years of organized football with > 3 years at the professional level. The former collegiate football players were required to have played >  6 years of organized football, with >  3 years at the college varsity level at similar high head impact playing positions. Controls were required to have no history of neurotrauma and no RHI exposure, no cognitive or psychiatric symptoms or diagnoses, and at least 2 years of post‐secondary education. Participants from all groups were excluded for the following reasons: (1) history of stroke or significant neurologic condition; (2) severe vision or hearing impairment; (3) inability to provide informed consent to participate; (4) currently clinically significant infectious, endocrine, metabolic, pulmonary, renal, hepatic disease, or cancer; and (5) body weight > 400 pounds (≈ 181 kg). Controls were excluded in case of (1) a history of neurotrauma; (2) cognitive impairment or psychiatric illness; and (3) a body mass index (BMI) < 24 kg/m^2^.

We used [18F]flortaucipir PET from the 180 former American football players and 60 controls from DIAGNOSE CTE. Importantly, in a previous study we found that tau‐PET standardized uptake value ratios (SUVR) among the American football players are on average below a diagnostic threshold, but significantly higher than controls in three brain regions (bilateral superior frontal, bilateral medial temporal, and left parietal).^26^ In addition, in a small sample of later deceased football players, we found a strong association between the *pre mortem* tau‐PET SUVR and *post mortem* p‐tau density in cortical and limbic regions.^27^ Moreover, we did not find group differences in amyloid‐β‐PET SUVR between the American football players and controls.^28^ Thus, the American football players likely do not have AD.

#### 2.1.2 Myelin water atlas

Information on typical MWF, an MRI‐based measure of cortical GM myelin and WM tract myelin levels were determined based on the publicly available Myelin Water Atlas.^24^ The atlas used myelin water imaging (MWI) – an MRI technique that quantifies myelin in‐vivo – in 50 healthy young adults.

#### 2.1.3 HCP

The HCP is a large‐scale multicenter study with the primary goal of mapping the human brain and its structural and functional connections using neuroimaging, among other techniques.^29^ We used diffusion MRI and resting‐state functional MRI (rs‐fMRI) from 100 healthy young adult participants from the HCP.^23^

**FIGURE 1:**
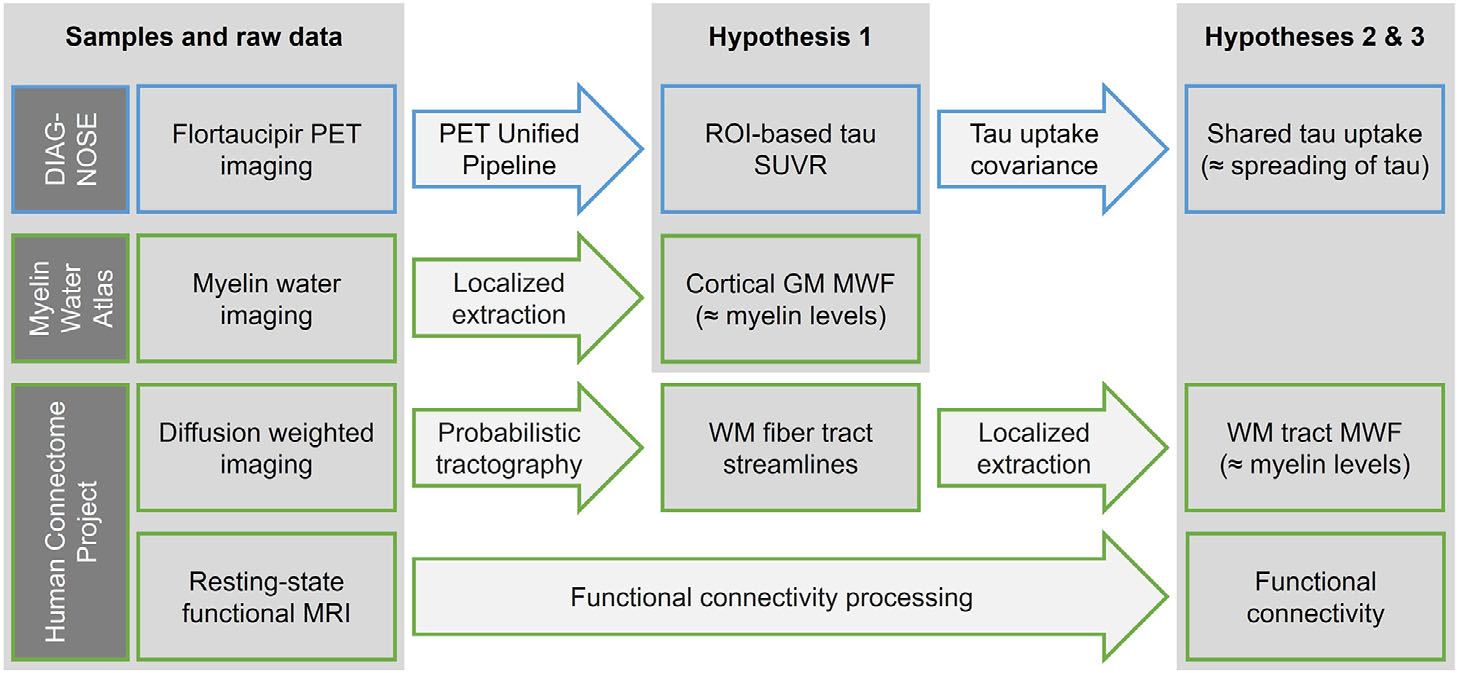
Overview of study samples, raw data, processing steps, and analyses. We used data on former American football players at risk for chronic traumatic encephalopathy as well as controls from the DIAGNOSE CTE research project (blue frames). We also used data from healthy young adult individuals from the Myelin Water Atlas and the Human Connectome Project (green frames). DIAGNOSE CTE, Diagnostics, Imaging, And Genetics Network for the Objective Study and Evaluation of Chronic Traumatic Encephalopathy; GM, gray matter; MRI, magnetic resonance imaging; MWF, myelin water fraction; PET, positron emission tomography; ROI, region of interest; SUVR, standardized uptake value ratio; WM, white matter

For an overview of study samples, raw data, processing steps, and analyses, see Figure 1.

### 2.2 Ethics approval

All studies were approved by the Institutional Review Boards of all involved sites and conducted in accordance with the Declaration of Helsinki. Written informed consent was obtained from all study participants before enrollment.

### 2.3 Image acquisition

#### 2.3.1 Flortaucipir PET

To obtain tau‐PET from the DIAGNOSE CTE study cohort, we used the [18F]flortaucipir tracer from Avid Radiopharmaceuticals (Philadelphia, PA, USA). The use of flortaucipir in this study was carried out through an Investigational New Drug (IND #131,391) from the United States Food and Drug Administration (U.S. FDA). The PET scans were acquired approximately 80–100 min after a bolus injection of 259 MBq (7 mCi) of the tracer on a PET/computed tomography (CT) scanner (GE Discovery 710 or Siemens mCT) in dynamic mode with 5‐min frames. All PET data were reconstructed with CT‐based attenuation correction and standard random and scatter corrections. Quality control and imaging calibration procedures were completed prior to study initiation by Invicro (Needham, MA, USA) to certify the scanners used in this study at each site. Of the 180 American football players and 60 control participants from the DIAGNOSE CTE research project, 11 American football players and three controls did not have [18F]flortaucipir PET data. In addition, the PET data of 12 American football players, four controls did not have matching MRI or the corresponding FreeSurfer outputs and were excluded, resulting in the final sample of 157 former American football players, and 53 controls.

#### 2.3.2 MWI

The Myelin Water Atlas is based on MWI, a technique to measure the signal from water trapped in myelin bilayers based on quantitative multi‐echo assessment of T2 relaxation.^24^, ^30^ The fraction of the myelin water signal relative to the total signal is referred to as MWF, which is a histopathologically validated MRI‐based approximation of myelin levels.^24^, ^30^ MWI was performed on a Philips Achieva 3 T MRI scanner (Philips, Best, The Netherlands). The sequence used a 3D gradient and spin echo (GRASE); 32 echoes; repetition time (TR), 1,000 ms; echo time (TE), 10 ms; SENSE, 2; 20 slices at 0.99 × 0.99 × 5 mm^3^ reconstructed to 40 slices at 1 × 1 × 2.5 mm^3^.^24^

#### 2.3.3 Diffusion MRI

Diffusion MRI from the HCP was acquired using a Siemens Magnetom Skyra 3T MRI scanner (Siemens Healthineers, Erlangen, Germany). The protocol used a spin‐echo echo planar imaging (EPI) sequence: TR, 5520 ms; TE, 89.5 ms; flip angle, 78°; 111 slices; 1.25 mm isotropic voxels; b values: 1000, 2000, and 3000 s/mm^2^.^31^

#### 2.3.4 rs‐fMRI

rs‐fMRI from the HCP was acquired using a Siemens Magnetom Skyra 3T MRI scanner (Siemens Healthineers, Erlangen, Germany). The protocol used a gradient‐echo EPI sequence: TR, 720 ms; TE, 33.1 ms; flip angle, 52°; 72 slices; 2.0 mm isotropic voxels.^23^, ^31^

### 2.4 Image processing

#### 2.4.1 Cortical tau‐PET uptake

[18F]Flortaucipir PET from the DIAGNOSE CTE study was processed using the PET Unified Pipeline (PUP).^32^ Briefly, PET images were smoothed to reach a common 8‐mm resolution, then between frame motion correction, target frame summation, and PET‐to‐MR co‐registration were performed sequentially and regional SUVR extraction based on the FreeSurfer generated anatomical ROIs with bilateral inferior cerebellar cortex as the reference region.

Cortical tau‐PET SUVR was extracted based on the Desikan–Killiany brain anatomy atlas^33^ with 62 regions of interest (ROIs; 31 per hemisphere). Of note, subcortical regions were excluded due to off‐target binding of flortaucipir.^27^ Correlated tau‐PET uptake was estimated by calculating the tau uptake covariance, that is, the Spearman correlation of tau levels between pairs of cortical regions.^19^ This resulted in a 62 × 62 matrix of covariance in tau and, hence, 62 × 61 / 2 data points for statistical analyses. Autocorrelations were set to zero and all correlations were Fisher‐z transformed. Tau‐PET uptake covariance is purported to be a proxy for spreading of tau, because the spatial covariance patterns of tau‐PET uptake resemble functional brain network topology, and it is posited that tau spreads via structural and functional connections.^17^ Previous research in AD using either tau‐PET uptake covariance or longitudinally assessed spreading of tau have both shown similar associations with functional connectivity.^17^, ^18^, ^19^

#### 2.4.2 Cortical myelin

The MWF maps from the Myelin Water Atlas^24^ were registered to T1‐weighted MRI images and then registered to the Montreal Neurological Institute (MNI) space. We applied the Desikan–Killiany atlas to the MWF to determine the regional myelin levels in the cortex as has been done previously.^20^

#### 2.4.3 WM tract myelin

Spatially normalized (i.e., to MNI space) and minimally preprocessed diffusion MRI images from the HCP^23^ were further processed using a multi‐shell multi‐tissue constrained spherical deconvolution and probabilistic tractography pipeline, as previously described.^17^ Based on the Desikan–Killiany atlas, we defined nodes and assigned the reconstructed fiber tract streamlines for each pair of ROIs. Next, we applied the above‐mentioned Myelin Water Atlas to the reconstructed streamlines and extracted the mean MWF along the streamlines between each of two ROIs as has been done previously.^20^ Based on the 100 resulting 62 × 62 matrices, one group average MWF matrix was calculated, resulting in 62 × 61 / 2 data points for statistical analyses similar to the tau covariance matrix.

#### 2.4.4 Functional connectivity

Spatially normalized (i.e., to MNI space) and minimally preprocessed rs‐fMRI images from the HCP^23^ were further processed using detrending, band‐pass filtering (0.01–0.08 Hz), despiking and motion correction, as previously described,^17^ before the Desikan‐Killiany atlas was applied. Next, for each of the 100 individuals, functional connectivity was calculated using Fisher‐z transformed Pearson‐moment correlations between all ROIs. Based on the 100 resulting 62 × 62 connectivity matrices, one group average connectivity matrix was calculated, resulting in 62 × 61 / 2 data points for statistical analyses similar the tau covariance matrix.

### 2.5 Statistical analysis

All statistical analyses were performed in R (RStudio version 2021.09.0). We used linear regressions to analyze (1) associations between typical cortical GM MWF and cortical tau‐PET SUVR in former American football players as well as controls; more specifically, we tested associations between the mean cortical GM MWF and the mean tau‐PET SUVR for each ROI of the Desikan–Killiany atlas and performed a spin test with 10,000 permutations to exclude spatial autocorrelation; (2) associations between typical WM tract MWF and tau‐PET uptake covariance (i.e., correlated tau deposition) in former American football players as well as group differences in this association (i.e., interaction of MWF by group on tau‐PET uptake covariance) and performed a quadratic assignment procedure (QAP) test with 10,000 permutations to exclude network interdependence; (3) associations between typical functional connectivity and tau‐PET uptake covariance (i.e., correlated tau deposition) in former American football players as well as group differences in this association (i.e., interaction of functional connectivity by group on tau‐PET uptake covariance) and another QAP test. We repeated analyses (2) and (3) after performing regional spread function (RSF) partial volume correction^34^ of the tau‐PET data. In addition, we performed additional sensitivity analyses, correcting the tau‐PET uptake covariance for the effects of age, BMI, years of education, imaging site, racial identity (binarized into White vs. Black or African American, American Indian or Alaska Native, Native Hawaiian or other Pacific Islander, and multiple races combined), binary apolipoprotein E ε4 (APOE4) carrier status, and cerebrospinal fluid (CSF) concentrations of amyloid β42/40. We report standardized *β*‐values. Following multiple comparison correction, *p*‐values < 0.05 were considered statistically significant.

Moreover, we added semi‐quantitative analyses to assess to what extent the tau‐PET SUVR pattern in American football players (already) aligns with the tau deposition pattern reported in CTE. Mean SUVRs were calculated for each Desikan–Kiliany atlas region, regions were divided into tertiles, and the highest and lowest tertiles were compared to literature‐defined CTE regions – typically including the superior, caudal middle, and rostral middle frontal, entorhinal, parahippocampal, insula, temporal pole, and inferior temporal regions.^7^, ^35^, ^36^ A region was considered a “match” if it fell within the top SUVR tertile and overlapped with CTE‐affected areas based on the seminal CTE literature,^7^, ^35^, ^36^ or within the lowest tertile and was not among the typically affected regions (and vice versa).

## 3 RESULTS

### 3.1 Sample characteristics

The 157 former American football players and 53 controls included were between 45 and 74 years old and male. For detailed information please see Table 1.

**TABLE 1:** Characteristics of former American football players and controls from the DIAGNOSE CTE research project.

Measure	Former American football players (*N* = 157)	Controls (*N* = 53)	*p*‐value
Age	Mean: 57.11, SD: 8.17 years	Mean: 59.47, SD: 8.62 years	0.076
Duration of education	Mean: 16.72, SD: 1.28 years	Mean: 17.43, SD: 3.34 years	**0.031**
Body mass index	Mean: 32.28, SD: 4.58 kg/m^2^	Mean: 30.70, SD: 4.44 kg/m^2^	**0.033**
Race	American Indian, Alaska Native: 0, Asian: 0, Black, African American: 54, Native Hawaiian, Pacific Islander: 0, White: 100, Multiple races: 3	American Indian, Alaska Native: 0, Asian: 0, Black, African American: 19, Native Hawaiian, Pacific Islander: 1, White: 33, Multiple races: 0	0.260
Apolipoprotein E ε4 carrier	Yes: 44, No: 105	Yes: 10, No: 40	0.192
Start age playing football	Mean: 11.01, SD: 2.83 years	–	–
Duration playing football	Mean: 16.01, SD: 4.24 years	–	–

*Note*: For derivation of sample size, please see section on image acquisition of flortaucipir PET.

Abbreviations: DIAGNOSE CTE, Diagnostics, Imaging, And Genetics Network for the Objective Study and Evaluation of Chronic Traumatic Encephalopathy; PET, positron emission tomography; SD, standard deviation.

The 100 healthy young adults from the HCP were 22–37 years old, 54% were female. The 50 healthy young adults from the Myelin Water Atlas were between 17 and 42 years, 50% were female.^24^ Information on racial identity was not available for these samples.

### 3.2 Cortical tau‐PET uptake in former American football player and cortical myelin

**FIGURE 2:**
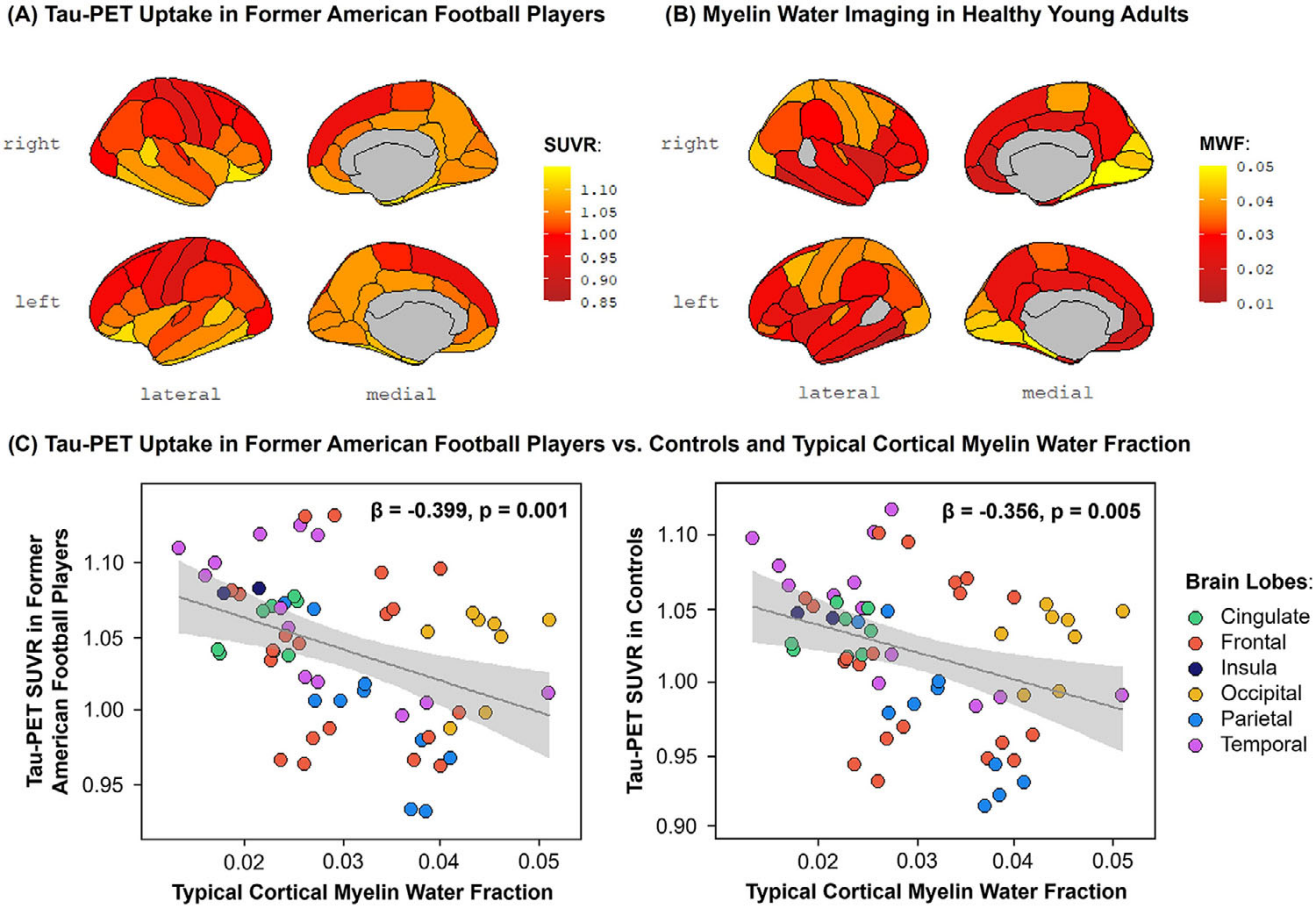
(A) Surface rendering of the average tau‐PET uptake in former American football players. Each cortical region displays the mean tau‐PET SUVR across the entire sample. (B) Surface rendering of average typical MWF) based on a healthy sample. Each cortical region displays the mean MWF across the entire sample based on a healthy sample. (C) Association between typical cortical gray matter MWF and cortical tau‐PET SUVR in former American football players (left) as well as controls (right), that is, on a group level. Every data point represents the mean tau‐PET SUVR and MWF for one brain region of the Desikan–Killiany brain anatomy atlas. Brain regions corresponding to each brain lobe are highlighted in the same color. MWF, myelin water fraction; PET, positron emission tomography; SUVR, standardized uptake value ratio

We found regionally varying tau‐PET uptake in former American football players (Figure 2 A) as well as region‐specific patterns of typical cortical GM MWF (Figure 2 B; subcortical regions excluded due to off‐target binding^27^). Typical cortical GM MWF was negatively associated with cortical tau‐PET SUVR in American football players (*β* = −0.399, *p* = 0.001; Figure 2 C *left*). Results did not change following the spin test. This association was less pronounced in controls (*β* = −0.356, *p* = 0.005; Figure 2 C *right*).

The tau‐PET uptake pattern in former American football players partly matches the brain regions typically affected by CTE (*matching*: high SUVR in lateral orbitofrontal, entorhinal, parahippocampal, medial orbitofrontal, temporal pole, inferior temporal, and insular regions, low SUVR in pre‐, para‐, postcentral, transverse temporal, superior parietal, and lateral occipital regions; *not matching*: low SUVR in superior, caudal and rostral middle frontal regions, high SUVR in fusiform and isthmus cingulate).^7^, ^35^, ^36^ Standard deviations in tau‐PET SUVR across all ROIs were between 0.055 and 0.108 in American football players, and between 0.050 and 0.090 in controls. For tau‐PET surface rendering in controls, see Figure S1.

### 3.3 Cortical tau‐PET uptake covariance in former American football players and WM tract myelin

**FIGURE 3:**
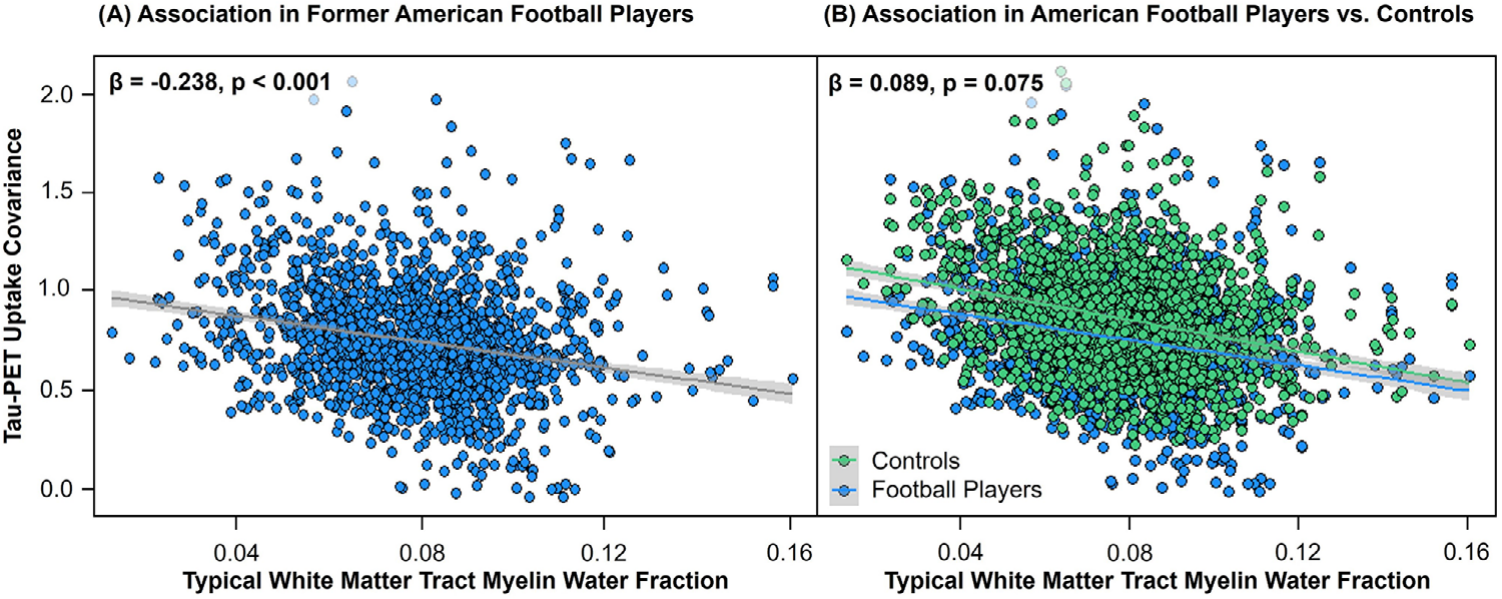
Associations between tau uptake covariance in American football players and typical myelin water fraction of connecting white matter tracts. (A) Association in American football players only; (B) interactive effect of group (American football players vs. controls from DIAGNOSE CTE) and myelin water fraction on tau‐PET uptake covariance. Every data point represents a connection between two brain regions of the Desikan–Killiany brain anatomy atlas (i.e., 62 × 61 / 2 data points). DIAGNOSE CTE, Diagnostics, Imaging, And Genetics Network for the Objective Study and Evaluation of Chronic Traumatic Encephalopathy; PET, positron emission tomography

We found a significant negative association between typical WM tract MWF and tau‐PET uptake covariance in former American football players (*β* = −0.238, *p* < 0.001; Figure 3 A). Results remained significant following the QAP test, partial volume correction (*β* = −0.227, *p* < 0.001), or sensitivity analyses, that is, when controlling the tau‐PET uptake covariance for the effects of age, BMI, years of education, imaging site, racial identity, APOE4 carrier status, and CSF amyloid β42/40 (*β* = −0.275, *p* < 0.001). The association was similar in controls, that is, there was no interactive effect of group and MWF on tau‐PET uptake covariance (*β* = 0.119, *p* = 0.075; Figure 3 B).

### 3.4 Cortical tau‐PET uptake covariance in former American football players and functional connectivity

**FIGURE 4:**
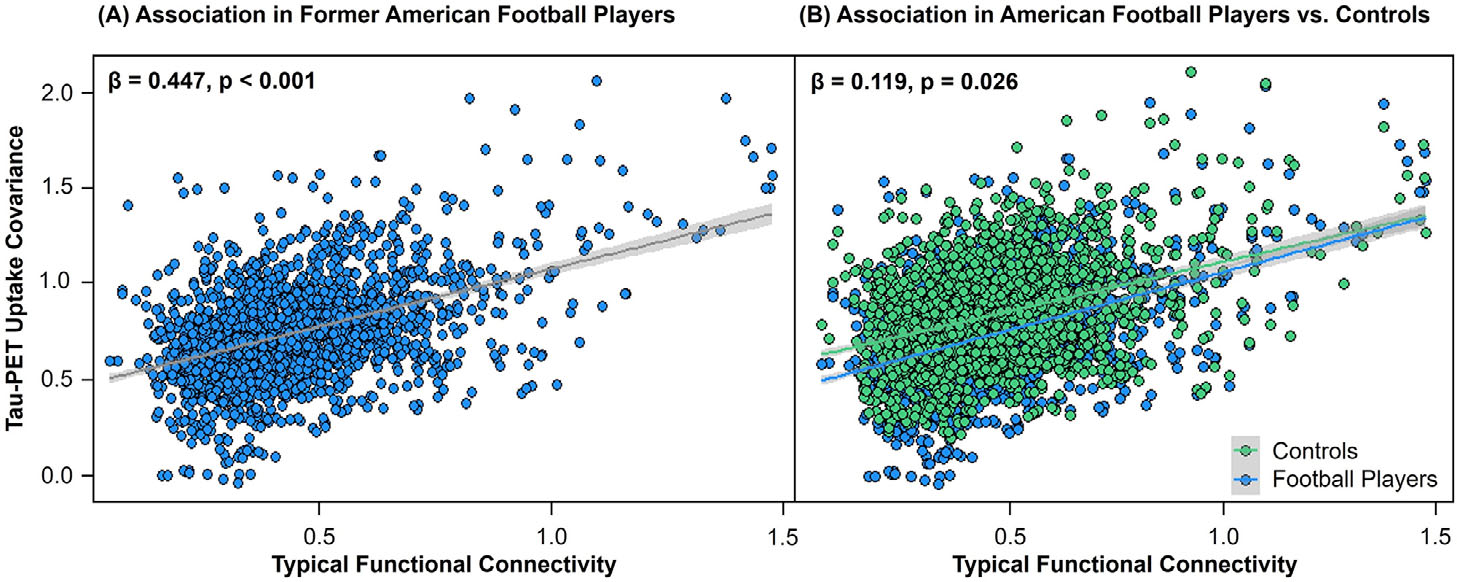
Associations between tau uptake covariance in American football players and typical functional connectivity of cortical regions. A) Association in American football players only; B) interactive effect of group (American football players vs. controls from DIAGNOSE CTE) and functional connectivity on tau‐PET uptake covariance. Every data point represents a connection between two brain regions of the Desikan–Killiany brain anatomy atlas (i.e., 62 × 61 / 2 data points). DIAGNOSE CTE, Diagnostics, Imaging, And Genetics Network for the Objective Study and Evaluation of Chronic Traumatic Encephalopathy; PET, positron emission tomography

We found a significant positive association between typical functional connectivity and tau‐PET uptake covariance in former American football players (*β* = 0.447, *p* < 0.001; Figure 4 A). Results remained significant following the QAP test, partial volume correction (*β* = 0.375, *p* < 0.001), or sensitivity analyses (*β* = 0.449, *p* < 0.001). The association was less pronounced in controls, that is, there was a significant interactive effect of group and functional connectivity on tau‐PET uptake covariance (*β* = 0.089, *p* = 0.026; Figure 4 B).

## 4 DISCUSSION

Findings from this study suggest that, in former American football players at risk for CTE, cortical regions with lower myelin levels may be more vulnerable to the deposition of tau, and cortical regions connected by WM fiber tracts with lower myelination may be more vulnerable to correlated deposition of tau. In addition, results from this study support the hypothesis that tau may spread via functional connections between cortical regions. Together, these results provide insight into what may underlie the distribution of tau deposition in individuals at risk for CTE, which may reflect a combination of processes, including age‐related changes, and potential disease‐related mechanisms specific to CTE or other forms of neurodegeneration.

We found that cortical regions that exhibit higher myelin levels as well as higher myelinated connections to other regions are less susceptible to pathologic tau deposition in individuals at risk for CTE. This is consistent with reports of similar associations between region‐specific myelination and lower tau‐PET deposition in AD.^20^ This association was also observable but slightly weaker in controls, possibly pointing toward a fundamental mechanism of regional susceptibility toward tauopathy including physiological aging, that is more pronounced in the American football players – many of whom likely did not have CTE at the time of the analyses or will never develop it. Thus, it was to be expected that the tau‐PET uptake pattern in American football players only partly matches the brain regions typically affected by CTE.

It has to be taken into account that flortaucipir shows limited affinity for the mixed 3R/4R tau isoforms characteristic of CTE and exhibits some off‐target binding (e.g., to monoamine oxidase, calcifications, or iron^37^). However, arguably, these effects are unlikely to substantially account for the consistent myelin‐ and connectivity‐related patterns observed here. Off‐target binding tends to occur in restricted subcortical or meningeal areas^37^ and would introduce random or region‐specific noise, which would weaken rather than strengthen the systematic associations we observed. In addition, a DIAGNOSE CTE study and a previous study on former American football players consistently found higher tau‐PET SUVR in the bilateral superior frontal, the bilateral medial temporal and left parietal regions that were associated with higher RHI exposure.^26^, ^38^ Likewise, tau‐PET can show inter‐regional correlations that can arise from global signal effects, partial volume effects, tracer spill‐over, and other scanner‐ or processing‐related factors. However, we include data from four PET scanners and results withstood spin test, QAP test, partial volume correction, and sensitivity analyses. Thus, despite limited specificity of the PET tracer and risk of autocorrelations, the spatial structure of tau‐PET uptake most likely at least partially reflects biologically meaningful variation in regional susceptibility.

The mechanisms behind potentially protective effects of higher cortical and WM tract myelin levels regarding region‐specific tau accumulation are poorly understood. The following arguments are thus speculative. One factor may be mechanical protection from myelin. More specifically, more layers of myelin may increase the mechanical resistance against tissue strain resulting from RHI, a known risk factor of CTE, by shielding the axons and absorbing mechanical forces.^39^, ^40^, ^41^

A second factor may be the role of oligodendrocytes in brain metabolism and microstructure. They allow for saltatory propagation of action potentials thereby reducing energy demand of neurons^42^ and provide energy metabolites to neurons via several specific transporters.^43^, ^44^ Damage or dysregulation of oligodendrocytes presents a risk factor for neurodegeneration.^42^ In fact, in CTE, multiple specific molecular and cellular alterations in myelin producing oligodendrocytes have been reported^45^ and hypothesized to result in alterations of the neuronal cytoskeleton and to increased susceptibility to tauopathy.^46^ In addition, myelin damage can activate tau‐specific kinases leading to hyperphosphorylation of tau^47^, ^48^ that may further trigger spreading of tau.^49^, ^50^

A third factor may be the role of oligodendrocytes in neuroinflammation. They regulate the activity of immune cells by secreting cytokines, chemokines, and receptors,^51^ and internalize and degrade myelin debris, which helps resolve inflammation.^52^ Neuroinflammation and dysregulation of these processes have been shown to play a crucial role in RHI and neurodegenerative diseases including CTE.^51^, ^53^

AD as well as most primary tauopathies are characterized by distinct spatial patterns of deposition and spreading of tau.^54^ In AD, Braak et al. have defined six stages of progressive tau pathology that appear to inversely recapitulate cortical myelogenesis during development.^46^, ^55^, ^56^ In CTE, McKee et al. have defined four stages^7^ that are distinct from the Braak stages. In our sample of former American football players, we found that the patterns of tau‐PET uptake are inversely associated with the region‐specific cortical myelin levels and only partly match the brain regions typically affected by CTE. In addition, as published previously, the American football players on average have sub‐threshold tau‐PET SUVR.^26^ This is likely due to the fact that, while the football players are at risk for CTE, not all players had CTE at the time of image acquisition. However, compared to controls, they have significantly higher tau‐PET SUVR in three brain regions that have been observed before (bilateral superior frontal, bilateral medial temporal, and left parietal cortex).^26^, ^38^ Moreover, in a subset of football players who later passed away and donated their brains, we found a strong association between the *pre mortem* tau‐PET SUVR and *post mortem* p‐tau density in cortical and limbic regions.^27^ Whereas CTE is frequently associated with other neurodegenerative diseases including AD, our sample of former American football players did not show higher amyloid‐PET uptake compared to controls,^28^ and results did not change when controlling for CSF amyloid β42/40. Thus, our findings are unlikely to be related to AD pathology.

We found that greater correlated tau deposition is associated with greater functional connectivity between brain regions in individuals at high risk for CTE. This association was more pronounced in former football players than in controls. Preclinical research that has shown that tau spreads via structural connections between cells with a prion‐like trans‐synaptic tau propagation.^13^ Higher neuronal activity and stronger functional connections may facilitate the release, uptake, and propagation of tau between brain regions.^14^ Moreover, phosphorylation of tau modulates its activity‐dependent release, with hyperphosphorylation leading to enhanced secretion, thus further supporting tau propagation.^57^ Additional potential explanations could be a shared genetic vulnerability, shared proteomic profile, or shared environmental influences between brain regions that may lie behind functional connections and could each influence the associations. Our findings are in line with preclinical studies showing that traumatic brain injury can induce transmissible tau species^16^ and with neuroimaging studies in other neurodegenerative diseases that report associations between fMRI‐assessed functional connectivity and PET‐assessed spreading of tau.^58^ More specifically, several studies report higher covariance in tau‐PET deposition between strongly connected brain regions compared to weakly connected regions in AD,^17^, ^18^, ^19^, ^20^ small vessel disease,^19^ cortico‐basal degeneration,^21^ and progressive supranuclear palsy.^21^

### 4.1 Limitations

There are several limitations to our results. (1) The DIAGNOSE study sample consists of former American football players who have been exposed to RHI and are thus at risk of CTE. It is unknown if the former American football players have CTE. (2) Flortaucipir PET is not suitable for diagnosing CTE. Several studies have pointed toward off‐target binding in flortaucipir PET.^37^, ^59^ This indicates limited specificity of the PET tracer to pathological tau protein depositions. *Post mortem* neuropathological analyses are needed to validate our findings. (3) The DIAGNOSE study sample consists of male former American football players that were active during the 1960s–1990s. Generalizability to females and other athlete populations is limited. (4) Whereas the former American football players were all males, around half of the participants from the Myelin Water Atlas and HCP were female. There is growing evidence of substantial differences in brain structure and function between females and males.^60^, ^61^, ^62^, ^63^ However, we found highly significant associations despite differences in sex; the effect may have been stronger in same sex individuals. Of note, the differences in age between former American football players and the samples from the Myelin Water Atlas and HCP are intended, as typical data requires younger and healthy samples. Future studies should include females exposed to RHI as well as sex‐specific environmental and biological measures such as daily activities or hormonal status. (5) We used cross‐sectional data from the DIAGNOSE CTE research project. Using the covariance of tau uptake as an approximation of spreading of tau is limited because it may be influenced by inherent PET signal correlations rather than disease‐related propagation mechanisms. Thus, no causal relationships can be determined, and future longitudinal studies are needed to further our understanding of spreading of tau in individuals at risk for CTE.

## 5 CONCLUSION

We demonstrate that cortical regions with lower typical myelin levels are more prone to tau deposition. In addition, correlated tau deposition is likely facilitated by WM tracts with lower typical myelin levels and by higher functional connectivity between brain regions. These mechanisms were evident in both former American football players and controls, suggesting fundamental determinants of regional susceptibility to tauopathy. However, the associations were more pronounced in former American football players, potentially contributing to their increased risk for CTE. Our findings provide a mechanistic framework linking structural and functional properties of the brain to tau propagation in RHI‐exposed individuals at risk for CTE. Longitudinal studies, including *post mortem* validation, are needed to confirm these pathways and their role in disease progression.

## CONFLICT OF INTEREST STATEMENT

C.H.A. consulted for Avion, CND Life Sciences, Jazz, and Precon Health. LJB is Editor‐in‐Chief of the Journal of Neuro‐Ophthalmology and is a paid consultant to Biogen (Cambridge, MA, USA). C.B. receives research support from the Ultimate Fighting Championship, Top Rank promotions, Haymon Boxing, Las Vegas Raiders, and Professional Bull Riders. He is a paid consultant for Aurora Concussion Therapy Systems, Inc. (St. Paul, MN). A.P.L. consulted for Agios, Biomarin, and Moncton MRI. He is a co‐founder of BrainSpec, Inc. J.L.C. has provided consultation to Acadia, Acumen, ALZpath, AnnovisBio, Artery, Axsome, Biogen, Bristol‐Myers Squib, Eisai, Fosun, GAP Foundation, Hummingbird Diagnostics, IGC, Janssen, Julius Clinical, Kinoxis, Lighthouse, Lilly, Lundbeck, LSP/eqt, Merck, MoCA Cognition, Novo Nordisk, NSC Therapeutics, Optoceutics, Otsuka, ReMYND, Roche, Scottish Brain Sciences, Signant Health, Simcere, sinaptica, and T‐Neuro pharmaceutical, assessment, and investment companies. E.M.R. is a compensated scientific advisor for Alkahest, Alzheon, Aural Analytics, Denali, Green Valley, Retromer Therapeutics, and Vaxxinity, and a co‐founder of ALZPath. RAS is a paid consultant to Eisai (Nutley, NJ, USA) and previously was a paid consultant to Biogen (Cambridge, MA, USA) and Lundbeck (Copenhagen, Denmark). He is a member of the Board of Directors of King‐Devick Technologies, Inc. (Chicago, IL, USA), and he receives royalties for published neuropsychological tests from Psychological Assessment Resources, Inc. (Lutz, FL, USA). He was a member of the Medical Science Committee for the National Collegiate Athletic Association Student‐Athlete Concussion Injury Litigation. I.K.K. receives funding for a collaborative project from Abbott Inc. She receives royalties for book chapters. Her spouse is board member at Siemens AG and stockholder of Siemens AG and Siemens Healthineers. The remaining authors have nothing to disclose. Author disclosures are available in the supporting information.

## CONSENT STATEMENT

All study participants provided written informed consent.

## Supporting information



Supporting Information

Supporting Information
