# Supplementary material for: Brain regional susceptibility to tauopathy in individuals at risk for chronic traumatic encephalopathy
Source: Alzheimers Dement. 2026 Jun 16;22(6):e71503. doi: 10.1002/alz.71503 (PMC13272103; doi:10.1002/alz.71503)
Supplement: Supplementary file 1 — Supporting Information [file ALZ-22-e71503-s002.docx]

**SUPPLEMENTARY MATERIALS**


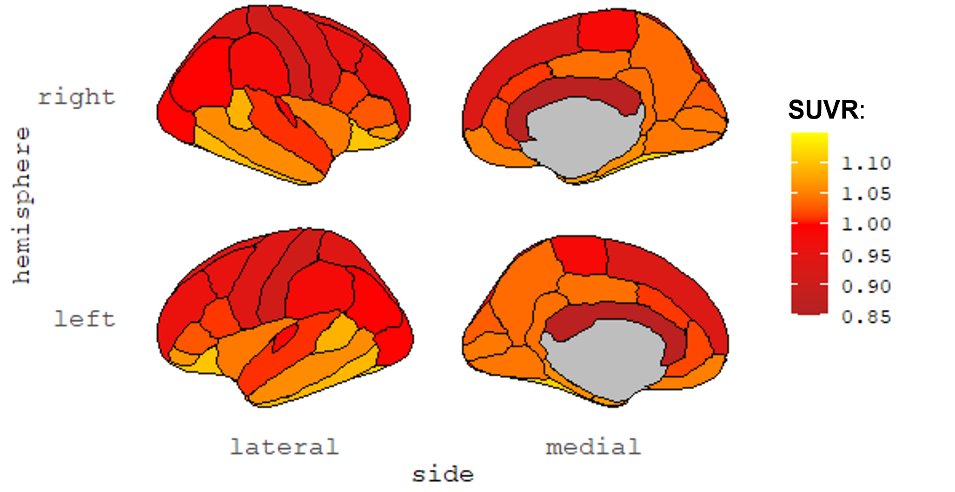


***Supplementary Figure 1***: Surface rendering of the average tau-positron emission tomography (PET) uptake in control participants without a history of exposure to repetitive head impacts from the DIAGNOSE CTE Research Project. Each cortical region displays the mean tau-PET standardized uptake value ratio (SUVR) across the entire group.
